# Assessing the measurement invariance of Free Will and Determinism Plus scale across four languages: a registered report

**DOI:** 10.1098/rsos.220876

**Published:** 2024-11-13

**Authors:** Siqi Duan, Chenghao Zhou, Qinglan Liu, Yixin Gong, Zenan Dou, Jingguang Li, Hu Chuan-Peng

**Affiliations:** ^1^ School of Psychology, Nanjing Normal University, Nanjing 210024, People’s Republic of China; ^2^ Faculty of Psychology, Autonomous University of Madrid, Madrid 28049, Spain; ^3^ Department of Psychology, New York University, New York 10003, USA; ^4^ GeseDNA Research Team, Beijing 100016, People’s Republic of China; ^5^ Faculty of Education, Monash University, Melbourne 3800, Australia; ^6^ School of Teacher Education, Dali University, Dali 671003, People’s Republic of China

**Keywords:** FAD-plus, measurement invariance, measurement equivalence, free will, cross-cultural research

## Abstract

Free will is assumed to be the core of an individual’s self-concept. Belief in free will has been studied extensively and was found to be correlated with many behavioural and psychological outcomes. Although developed and validated in the West, the Free will and Determinism Plus (FAD-Plus) scale has been translated, used, and interpreted as a measurement of free will beliefs in multiple cultures. However, the cross-cultural measurement invariance of FAD-Plus has not been examined. Given the cultural differences in understanding the concept of ‘free will’, items of FAD-Plus may have different interpretations in different cultures, which may compromise its cross-cultural measurement invariance. To provide empirical evidence for the lack of cross-cultural measurement invariance, we collected data in China and analyzed these data together with open datasets of FAD-Plus in three other languages: Japanese, French and English. We only found partial measurement invariance between the Chinese and English datasets, as well as the Japanese and English datasets. These results provided the first assessment of cross-cultural measure invariance of FAD-Plus. We discussed the potential implications of the current study for future studies in the field.

## 1. Introduction

Free will is the core of an individual’s self-concept as a subject capable of rational, independent thinking, and decision-making [1]. The lay belief in free will, that people have the capacity to act freely or could have chosen to do otherwise [2], has been studied extensively in psychology [1,3–5]. Belief in free will is the general belief of lay people that human behaviour is free from internal and external constraints across situations for both self and others [4]. It is a unique psychological construct as it focuses on the capacity for choice and constraints from both the individual and environment (see Feldman [4] for detailed discussion). Previous studies revealed that belief in free will is associated with a variety of psychological/behavioural outcomes, e.g. life satisfaction, across different cultures [6–9].

As the foundation of quantitative studies, researchers have developed multiple scales to measure belief in free will. As the ‘free will notion … is the heart of Western religious, philosophical, and legal understandings of moral responsibility’ [10], it is not surprising that scales of belief in free will were all developed in the West. Since the first measurement of free will beliefs [11], various scales of belief in free will had been developed. For example, a 19-item scale named the Free Will-Determinism Scale with a three-factor structure, religious-philosophical determinism, psychosocial determinism and libertarianism, from Stroessner and Green [12]; and a 29-item scale named Free Will Inventory (FWI) [13], that measures the strength of people’s belief in free will, determinism, dualism and responsibility.

The scales of free will beliefs, however, are used in different cultural contexts. In some cases, researchers compared beliefs in free will across different cultures based on the observed scores [14,15]. The most widely used scale is the Free will and Determinism Plus (FAD-Plus) scale, which was developed by Paulhus and Carey [16]. FAD-Plus inherited the compatibilism from Stroessner and Green [12] and Rokas [10]. It includes four dimensions: free will, scientific determinism, fatalistic determinism and unpredictability. The free will sub-scale measures people’s belief in free will, which has items such as ‘People have complete control over the decisions they make’. The scientific determinism sub-scale measures the belief in scientific causality, with items such as ‘People’s biological makeup determines their talents and personality’. Fatalistic determinism measures the belief in fate, with items like ‘I believe that the future has already been determined by fate’. The scientific determinism and fatalistic determinism sub-scales are distinguished by the type of deterministic thinking [16]. The fourth sub-scale, unpredictability, measures the belief in the unpredictable and unknowable, which is a conceptual dimension independent of free will, belief or determinism [16].

FAD-Plus has already been translated into at least five different languages [17–20], including several Chinese versions (e.g. [21,22]) and two Japanese versions [19,23]. These translated versions of FAD-Plus are vital for comparing and exploring the associations between free will beliefs and a variety of psychological outcomes across different cultures [18–22,24]. For example, Paulhus and Carey [16] found that the Free will dimension has a strong positive correlation with Extraversion and the Fatalistic Determinism has a strong positive correlation with Neuroticism in a Canadian sample. In Goto [19]’s study, the Free will dimension showed a strong positive correlation with patience in a Japanese sample. An Italian study found that Scientific Determinism has a strong correlation with Self-Representations, Extraversion and Other Representations [18]. After reviewing 36 studies from regions including the United States, Singapore, Hong Kong, India, Turkey and Germany, Lam [1] found that, overall, people believed in free will across regions. Lam [1] also found that beliefs in free will mean different actions under different cultural backgrounds. However, like other measurements of belief in free will, FAD-Plus was also developed in English based on a narrow sample of population, i.e. North American or European undergraduates in their 20s [1]. So far, no study has compared these translated versions of FAD-Plus to the English version in terms of measurement invariance.

Measurement invariance (or measurement equivalence) is a psychometric notion that a scale or task measures the same concept across different groups, situations and times [25–27]. Without testing measurement invariance, we cannot directly compare the scores or latent constructs of a scale across different cultures since they may measure different psychological concepts [28,29]. Milfont and Fischer [29] identified four levels of measurement invariance. The first and lowest level is configural measurement invariance, which means the scale shares the same structure (i.e. the number of latent constructs) across each measured group. For FAD-Plus, configural invariance means that data from different languages should exhibit the same four-dimensional structure. The second level is metric invariance (also called weak invariance), which means the scale has the same factor loadings for each item. If this level of MI holds for FAD-Plus, it means that not only do FAD-Plus across different languages share a four-dimension structure, but items’ weight or loading of each dimension are similar. A higher level, scalar invariance (or strong invariance), requires equal item intercepts, indicating equal mean scores for each item across different groups. At this level of measurement invariance, researchers can compare the means of latent variables (e.g. four dimensions of FAD-Plus) across different groups. The fourth and final level, strict invariance (also known as error variance invariance or full uniqueness measurement invariance), means that item residual variances are equal across groups. This level of measurement invariance is not essential for comparing the means of the scale across different groups [30]; thus, we will not include this level of measurement invariance in our analyses.

We infer that even the metric invariance (i.e. weak invariance) may not hold across the FAD-Plus scale in different languages due to the following reasons. First, the phrase ‘free will’ is not a native concept in many non-Western languages [31]. As mentioned above, the concept of free will is deeply rooted in Western cultural tradition and imported to other cultures. For example, in Chinese and Japanese languages, the phrase ‘free will’ (自由意志) is a translated philosophical jargon that coined two existing words, ‘free’ (自由) and ‘will’ (意志). In such cases, ‘free will’ often functions as an academic or technical term, which many people, particularly those without higher education, may struggle to understand or may misinterpret. Moreover, in China, ancient philosophers expressed ideas that are similar to ‘free will’ but with different connotations. For example, Confucius, one of the most important ancient philosophers in China, has a famous saying: ‘七十而从心所欲不逾矩’, which was translated as ‘at seventy, I followed what my heart desired without overstepping the line’ [32]. Confucius highlighted an ideal state in which the will of a decent person (君子) is naturally consistent with the norms, instead of against external/internal constraints. This tradition may cause different interpretations of items such as ‘people have complete free will’. For a Confucian, ‘complete free will’ might refer to achieving that ideal state, a meaning that might not occur to a responder from the West. Another difference may exist in the interpretation of ‘fate’: while in the Western world, fate is usually associated with the will of God, in Buddhism’s view, fate is part of the laws of nature (自然法则). These differences may cause different interpretations for items such as ‘Fate already has a plan for everyone and I believe that the future has already been determined by fate. These cultural and societal factors may lead to further differences in the interpretation of items within the FAD-Plus. These differences, in turn, may cause different loadings of items on latent factors, or worse, cause different configural structures of FAD-Plus. In other words, these cultural differences will result in non-invariance even for the lowest level. Furthermore, measurement invariance is not only important for comparisons between countries/regions but also for sub-groups that use the same language or within the same region. For example, education may play an important role in free will belief as higher education is usually associated with more knowledge about science. Thus, it is important to compare the measurement invariance of FAD-Plus across sub-groups with different educational levels (e.g. different Chinese samples).

The unknown status of measurement invariance makes it difficult to interpret the results from cross-cultural comparison of belief in free will (see similar view in other cross-cultural studies [33,34]. If the cross-language measurement invariance of FAD-Plus does not hold, researchers would need to reconsider what FAD-Plus measures in different cultures, how to conceptualize this construct in a global context, and how to design studies to measure it, such as through a multi-national collaboration project (e.g. [35]). The present study aims to assess the measurement invariance of FAD-Plus across four different languages. We collected a new dataset in China using an improved version of the Chinese FAD-Plus and retrieved open data from three other languages: English, French and Japanese. The Chinese data were collected from a diverse Chinese sample, using a Chinese translation of FAD-Plus that follows the standard translation procedure. Data for FAD-Plus in other languages was retrieved online, such as the English version [36–38], or generously shared by the authors, e.g. the Japanese [19,23] and the French version [17]. This project provided insights into measuring free will beliefs across different cultures.

## 2. Method

### 2.1. Data

The Chinese data were collected by three Chinese teams (SD, CHZ, YG and HCP; QLL and ZD; JL) immediately after Stage 1 of the Registered Report was accepted in principle, and each team aimed to recruit at least 400 valid participants. One team collected data from 572 high school students, but only 356 passed the validation checks. The other two teams collected valid data from 455 and 437 participants. The sample sizes exceeded 200 per group, as recommended by Koh and Zumbo [39] for measurement invariance analysis, and this was also the case for our sub-group analysis between different sites. Participants have a wide range of ages, educational levels and locations. The three teams collected data based in three cities: SD, CHZ, YG and HCP collected data in Nanjing (in South China), QLL and ZD are based in Beijing (in North China) and JL is based in Dali (in Southwest China). These three teams targeted different samples: college students, general young adult internet users who are undertaking online meditation training, and high school students, respectively. We collected socio-demographic variables, including gender, age, educational level, monthly family income and foreign experience. These variables were used to assess the diversity of our samples. This study was conducted according to the Declaration of Helsinki. The research protocol has been approved by the Institute Review Board of Nanjing Normal University (No. NNU202110002). All participants were clearly informed, and their consent was obtained before collecting any data. See electronic supplementary material, S1.3, for more details about the data collection procedure.

Datasets of FAD-Plus in English, French and Japanese were retrieved from available sources. As for our English datasets, we combined data from three studies on OSF: Earp *et al.* [36], which included four studies (1A, 1B, 1C and 2) conducted in England and the US that examined the relationship between belief in free will and humility; Post and Zwaan [38], which studied the value of believing in free will in North America and Netherlands; and Nadelhoffer *et al*. [37], which explored cheating behaviour with the belief in free will from the US. In total, the English datasets comprised 3256 participants with a mean age of 28.85 (s.d. = 15.15). The French data were collected in Belgium by [17], who examined the reliability of the French translation of FAD-Plus with a sample size of 904 and a mean age of 26.29 (s.d. = 8.68). The Japanese data were collected using two versions of the translation [19,23], which we have separated into two datasets: the early version with 3272 participants and a mean age of 38.15 (s.d. = 10.62); the later version included 800 participants with a similar mean age.

### 2.2. Material

#### 2.2.1. The Free will and Determinism Plus (FAD-PLUS) Scale

As mentioned above, the FAD-Plus was developed by Paulhus and Carey [16]. This scale consists of four subscales: Free Will (7 items), Scientific Determinism (7 items), Fatalistic Determinism (5 items) and Unpredictability (8 items). Each item is rated on a 5-point Likert scale: ‘1 = strongly disagree, 5 = strongly agree’. No item needs reverse scoring.

The Chinese translation of FAD-Plus used in the current study was an improved version based on three earlier translations [9,21,22,40]. More specifically, this version resolved the inconsistencies between three previous translations and was re-translated according to the translation and back-translation method (e.g. ITC [41]). For additional details on the translation process, see electronic supplementary material, S1.3 Procedure of Chinese Data Collection and https://osf.io/t7p43/.

The French version of FAD-Plus was translated following the back-translation procedure; see Caspar *et al*. [17] for more details. The Japanese version of FAD-Plus has two different sub-versions, both following the back-translation procedure; please see Goto [23] for more details. The English version used the original FAD-Plus by Paulhus and Carey [16].

#### 2.2.2. The Big Five Inventory

Similar to the original study by Paulhus and Carey [16] and Caspar *et al*. [17], we validated the FAD-Plus using the Big Five personality traits, which include Conscientiousness, Agreeableness, Openness, Neuroticism, and Extraversion. The original study found that the Free Will subscale of the FAD-Plus was positively correlated with the Extraversion, Neuroticism, and Agreeableness subscales of the Big Five [16].

In the new Chinese data, we used the Big Five Inventory−2 (BFI−2) as a measure of the Big Five personality. BFI−2 is a revised version of the Big Five Inventory [42]. It scores on a 5-point Likert scale. Participants were asked to rate their agreement with each statement on a scale from ‘1 = strongly disagree’ to ‘5 = strongly agree’. The Chinese version of the BFI−2 was translated by Zhang *et al*. [43].

Data of the BFI were also available in French language [44]; see [17] for details. However, BFI data were not available in Japanese datasets and English datasets.

#### 2.2.3. Locus of control

Locus of control was also used to test the criterion validity of the FAD-Plus in the original study by Paulhus and Carey [16]. This study reported that the Free Will subscale was positively correlated with internal control, the Fatalistic Determinism subscale was correlated with aspects of external control, and the Scientific Determinism subscale was correlated with both internal and external control.

We used the Chinese version of the multi-dimensional locus of control (MLOC) inventory, which was translated from Levenson [45] and has demonstrated good psychometric properties in Chinese samples [46]. The MLOC consists of 24 items, with each of its three dimensions represented by eight items: internality, control by powerful others and control by chance forces. It is scored on a 6-point Likert scale, ranging from −3 (strongly disagree) to 3 (strongly agree). However, due to an implementation error, the rating scale of the MLOC in the first test used a 7-point Likert scale instead of the original 6-point scale. This error was corrected before the retest. Consequently, the test-retest reliability of the MLOC was not calculated, and we only used data from the retest.

In the Japanese datasets, data on locus of control was collected, see Goto [23] for more details. Note that the Japanese locus of control scale is a 7-point Likert questionnaire with seven items. These seven items constitute two subscales: external and internal control. There was no data of locus of control data in the French and English datasets.

### 2.3. Procedure

We collected the Chinese data online powered by Qualtrics XM. The scales were presented in the following order: FAD-Plus, BFI−2 and MLOC (see deviation). To calculate the test-retest reliability, we re-tested a subset of participants, with an interval of approximately four weeks. The data collection procedures in other languages had been described in the original papers and are not repeated here.

### 2.4. Data analysis

We used R (4.3.1) and other R packages to analyze the data. All data were preprocessed using tidyverse (1.3.0 [47]). The following R packages were used in our analysis: CTT (2.3.3 [48]), dplyr (1.0.2 [47]), lavaan (0.6–7 [49]), psych (2.0.12 [50]), semPlot (1.1.2 [51]), semTools (0.5–3 [52]), NNLM (0.4.3 [53]) and RcppML (0.5.6 [54]). All the scripts are available at: https://github.com/Chuan-Peng-Lab/FAD_Plus_Stage2.

#### 2.4.1. Descriptive

See table 1 for the descriptions of Chinese datasets and all variables. The correlations between items and their corresponding dimensions can be found at https://osf.io/n5h7t/.

**Table 1.** Information on distinct source of data from different language datasets.

			FAD-Plus						BFI			LOC		
dataset ID	subdataset	FAD-plus version	sample size (N)	mean age	gender	educational attainment	family income (per month, RMB)	foreign experience	sample size (N)	mean age	gender	sample size (N)	mean age	gender
CHN	dataset 1 (By SD, CHZ, YG, and HCP in Nanjing)	CHN	437	21.91 ± 2.28 NA = 2	*F* = 223 *M* = 214	*n*_1_ = 1; *n*_3_ = 4; *n*_4_ = 11; *n*_5_ = 323; *n*_6_ = 96; *n*_7_ = 2	mean = 24 106 median = 12 000	yes = 39 no = 398	437	21.91 ± 2.28 NA = 2	*F* = 223 *M* = 214			
	dataset 2 (By QLL and ZD in Beijing)	CHN	455	31.00 ± 8.38	*F* = 378 *M* = 77	*n*_2_ = 2; *n*_3_ = 22; *n*_4_ = 27; *n*_5_ = 248; *n*_6_ = 129; *n*_7_ = 27	mean = 34 255 median = 15 000	yes = 133 no = 322	455	31.00 ± 8.38	*F* = 378 *M* = 77			
	dataset 3 (By JL in Dali)	CHN	356	16.94 ± 1.35	*F* = 229 *M* = 127	*n*_3_ = 347; *n*_5_ = 2; n_6_ = 7	mean = 7214 median = 5000	yes = 4 no = 352	356	16.94 ± 1.35	*F* = 229 *M* = 127			
	retest-dataset (By SD, CHZ, YG, & HCP in Nanjing)	CHN	188	22.10 ± 2.03	*F* = 80 *M* = 108	*n*_3_ = 1; *n*_4_ = 3; *n*_5_ = 137; *n*_6_ = 46; *n*_7_ = 1	mean = 14 914 median = 12 000	yes = 17 no = 169	188	22.10 ± 2.03	*F* = 80 *M* = 108	188	22.10 ± 2.03	*F* = 80 *M* = 108
ENG^a^		ENG	3256	28.85 ± 15.15 NA = 28	*M* = 1733 *F* = 1478 NA = 276				—	—	—	—	—	—
FRN^b^		FRN	904	26.29 ± 8.68 NA = 7	*M* = 579 *F* = 325				195	25.73 ± 8.49	*M* = 138 *F* = 57	—	—	—
JPN_1^c^		JPN_v1	3727	38.15 ± 10.62 NA = 2925	*M* = 246 *F* = 546 NA = 2935				—	—	—	802	38.15 ± 10.62	*M* = 246 *F* = 546 NA = 10
JPN_2^d^		JPN_v2	800	38.16 ± 10.62	*M* = 245 *F* = 545 NA = 10				—	—	—	800	38.16 ± 10.62	*M* = 245 *F* = 545 NA = 10

Note: The rating scale for MLOC was incorrect in our first test at all three Chinese sites due to an implementation error (see procedure). Therefore, we did not include the first test data for LOC. However, all this data was recorded and is available at https://osf.io/utrmv/.

Levels of educational attainments:1: primary school or less; 2: middle school or equivalent; 3: high school or equivalent; 4: some college, vocational school after high school; 5: college graduate, with bachelor’s degree or in college/university; 6: master, with master’s degree or in a master program; 7: doctor and higher, with doctor degree or in the PhD program.

^a^ ENG^1^ data source: [36–38]

^b^ FRN^2^ data source: [17]

^c^ JPN_1^3^ data source: [19,23];

^d^ JPN_2^4^ data source: [23]

#### 2.4.2. Reliability

Based on the classic test theory, we calculated the internal reliability of subscales and the whole test for FAD-Plus, with Cronbach’s alpha and McDonald’s omega coefficients as indicators. The test-retest reliability was reported based on data from participants who completed the test twice. Cronbach’s alpha and Mcdonald’s omega were calculated using the R package *psych* (2.0.12 [50]).

#### 2.4.3. Validity

As for the validity, we tested the construct and criterion validity. While measurement invariance is also a facet of validity, it was reported separately to emphasize its importance.

##### 2.4.3.1. Construct validity

Using the original four-factor model from Paulhus and Carey [16], we tested the model fit and extracted the loadings of items. We compared the constructs from different languages’ datasets. Specifically, we evaluated the relationship between each item and its corresponding dimension across distinct datasets.

##### 2.4.3.2. Criterion validity

Similar to Paulhus and Carey [16], we estimated criterion validity by calculating correlations between the four dimensions of FAD-Plus and five dimensions of the BFI, as well as with the subscales of the MLOC (electronic supplementary material, table S2).

To compare these correlations with the original study, we employed a bootstrap sampling approach [55]. This involved generating a distribution of correlation coefficients (*r*-values) based on 5000 bootstrap samples for each pair of variables. We randomly selected observations with replacements from the newly collected data and calculated the correlations that index the criterion validity. The resulting distribution of *r*-values allowed us to estimate the mean *r*-value and its 95% confidence interval. More importantly, we can infer whether each correlation significantly differs from the originally reported *r*-value in Paulhus and Carey [16]. More specifically, if the original *r*-value falls outside the 95% confidence interval of the corresponding bootstrap distribution, we infer that the correlation from the new data is statistically different from the original correlation. Additionally, we calculated the *p*-value for the observed correlation coefficient based on its position within the bootstrap distribution.

### 2.4.4. Measurement invariance

**Figure 1.**
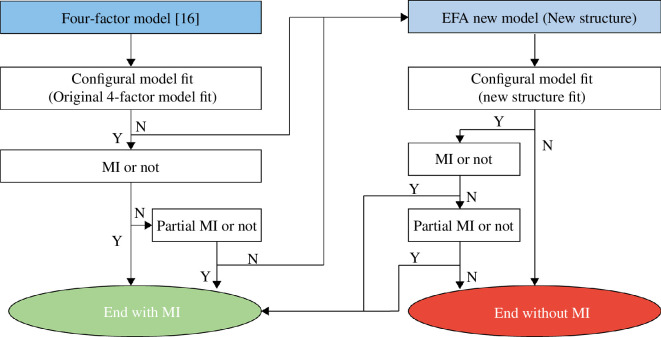
Procedures of detection of measurement invariance (MI) in two different language datasets.

For measurement invariance, we compared different non-English datasets with the English ones. Besides the traditional multi-group confirmatory factor analysis (CFA) method [56], we also included a partial metric [57] since weak measurement invariance might not hold. The analytical workflow for measurement invariance was planned and carried out as in figure 1. Firstly, we tested Paulhus and Carey’s [16] original four-factor model in all datasets. Once the original model fit the datasets, we continued to test the measurement invariance with the traditional CFA multi-group method. In the event of failing to achieve metric invariance, we proceeded to test partial measurement invariance instead.

Although we obtained partial measurement invariance for the FAD-Plus data between the three non-English datasets and the English dataset, we employed the strategy from Iurino and Saucier [58]. Specifically, we created a new dataset by randomly selecting half the data in each of the four different language datasets. This new, multi-language dataset was used for exploratory factor analysis (EFA, see electronic supplementary material, S3.3). Refer to figure 1 for the workflow of our measurement invariance analysis.

In addition to comparing the measurement invariance across different languages, we also examined measurement invariance among different groups that use the same language. We compared the measurement invariance of different groups in our Chinese samples because we collected relatively diverse datasets representing different sub-groups of the Chinese population (college students, adults outside the university community and high school students). For data from other languages, for example, two Japanese versions, their measurement invariance was also explored and reported.

Besides the multi-group CFA, we conducted additional analysis to assess measurement invariance using item response theory (IRT) and non-negative matrix factorization (NMF). See supplemental materials for more details and results.

### 2.5. Disclosure

We submitted this project as a registered report and the current document is at Stage 2 of the registration. The in-principle accepted Stage 1 protocol is available at https://osf.io/umhvp.

This project is based on our first attempt to translate FAD-Plus into Chinese and examine the psychometric properties of the Chinese version [22]. All de-identified data and scripts are available at https://osf.io/utrmv/. Also, we reported additional methodological details, results, and plots in the ‘Supplementary analyses and results for measurement invariance’ (§2) of the electronic supplementary material. Materials used for the data collection in China can be accessed at https://osf.io/t7p43/. To maintain full transparency, we have made submission records, reviewers’ comments, response letters and decision letters available in the ‘submission_history’ folder on https://osf.io/utrmv/.

#### 2.5.1. Deviations from the Stage 1 protocol

We documented all deviations from the Stage 1 protocol throughout the data collection process for transparency. All these deviations were unanticipated and did not significantly affect the strength of the evidence. Firstly, one team collected fewer valid participants than planned. The plan was for each team to recruit at least 400 valid participants following Stage 1. However, one team recruited 356 valid participants from a survey of 572 high school students. Due to practical reasons (i.e. authors’ agreement with local high schools), we were unable to collect more data after the initial round of data collection. Despite these deviations, with an overall sample size exceeding 1200, the data from the three cities (Nanjing, Beijing and Dali) met the requirement for measurement invariance analysis and provided a diverse demographic range, which included variations in age, education and location.

Secondly, the data were collected via Qualtrics XM instead of JsPsych hosted on GitHub. This was due to unstable access to GitHub in some regions of mainland China. This change caused an unexpected implementation error: the order of testing for FAD-Plus, BFI−2 and MLOC deviated from our initial randomized design. We ensured that all our participants experienced the same order of questionnaire materials, and usually, the order of tests did not lead to significant consequences (e.g. [59]).

Thirdly, a 7-point scale was mistakenly used for the Chinese MLOC scale, instead of the intended 6-point Likert scale. We corrected this error in the Nanjing retest sample (with a retest rate of 60.64%). Only the MLOC retest data were analyzed in the main text. We also provided correlational analyses between four factors of FAD-Plus and both the 6-point and 7-point scales to ensure transparency (see electronic supplementary material, table S1).

Fourthly, we used a bootstrap method to compare the correlation values in the original study and the correlations calculated from the new datasets. These correlations served as indices of criterion validity for FAD-Plus. We employed the bootstrap method because it allowed us to rigorously evaluate whether the observed correlations in the new datasets significantly differ from the original reported values. These results provided additional evidence regarding the cross-cultural variability in the relationships between the dimensions of the FAD-Plus and the personality measures (BFI and MLOC).

## 3. Results

### 3.1. Descriptive

Data from Chinese participants were collected by three Chinese research teams. In total, 1248 participants were included, with 830 females and 418 males, a mean age of 23.81 years and a standard deviation of 7.85 years. Detailed descriptions of each dataset can be found in table 1. Participants in Dataset 2 were from a diverse age range and educational levels. Dataset 3, as planned, was collected among high school students and had a lower mean age than the other two datasets. Dataset 1 was from a university community, and it included both undergraduate students and graduate students. Only participants in Dataset 1 took the retest.

When compared to datasets in other languages, the newly acquired Chinese data generally exhibits a satisfactory sample size and a mean age consistent with the English and French datasets (table 1).

### 3.2. Reliability

Internal reliability for the subscales and the entire test of FAD-Plus was assessed using Cronbach’s alpha and McDonald’s omega. In comparison with the original α values reported by Paulhus and Carey [16], all datasets showed satisfactory reliability. The test-retest reliability values, calculated using Pearson’s correlation coefficients for each dimension, are also listed in table 2.

**Table 2.** Cronbach’s alpha and McDonald’s omega with different datasets in distinct dimensions.

	original (α)	CHN (α/ω)	CHN test-retest (*r*)	ENG (α/ω)	FRN (α/ω)	JPN_1 (α/ω)	JPN_2 (α/ω)
whole	-	0.794/0.803		0.803/0.861	0.714/0.694	0.825/0.838	0.790/0.830
FD	0.82	0.751/0.809	0.684	0.860/0.889	0.739/0.782	0.753/0.798	0.711/0.781
SD	0.69	0.671/0.785	0.653	0.769/0.825	0.597/0.677	0.678/0.748	0.641/0.741
UP	0.72	0.745/0.807	0.613	0.791/0.847	0.709/0.762	0.786/0.824	0.792/0.853
FW	0.70	0.749/0.825	0.745	0.842/0.891	0.710/0.782	0.663/0.753	0.660/0.762

### 3.3. Validity

#### 3.3.1. Construct validity

The results of the CFA indicated that all language datasets fit the four-factor model well. The loadings are presented in electronic supplementary material, table S5 in the electronic supplementary material.

#### 3.3.2. Criterion validity

Correlational analyses were performed between the dimensions of FAD-Plus and the factors of the BFI for the Chinese and French datasets, respectively. Additionally, correlations between the FAD-Plus and MLOC in the Chinese retest dataset were computed. The Japanese dataset was not analyzed due to the use of a different LOC scale.

**Figure 2.**
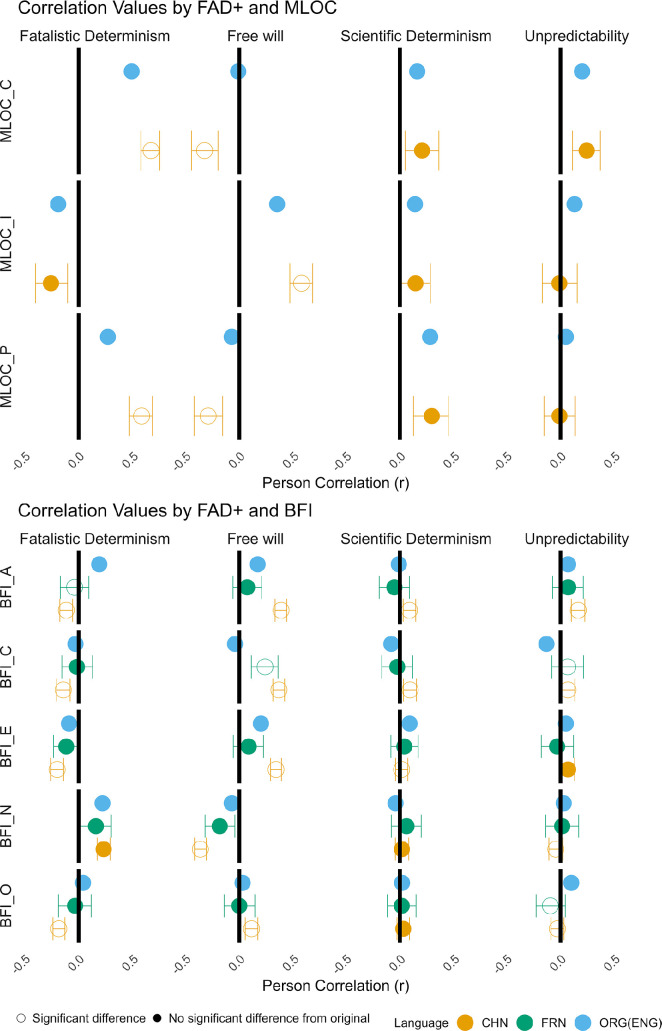
Correlations between four dimensions of the FAD-Plus and three dimensions of the multidimensional locus of control (MLOC) (upper) and five dimensions of the Big Five Inventory (BFI) (lower). ORG: The original reported results (English); CHN: Mean r-value after 5000 bootstraps from Chinese newly collected data; FRN: Mean *r*-value after 5000 bootstraps from the open-access data from French language community by [17]. The error bars represent 95% confidence intervals. Filled dots indicate that the original *r*-value falls within the 95% bootstrap CI, while unfilled dots mean the *r*-values does not fall within the 95% bootstrap CI, i.e. a significant difference from the original (ORG) results.

For the FW (Free Will) dimension, we observed that all correlations between FW and both MLOC and BFI differed from the original study. For the correlations that were significant in the original study (MLOC_I, BFI_A and BFI_E), the Chinese dataset exhibited higher correlations. For those originally non-significant correlations (MLOC_C, MLOC_P, BFI_C, BFI_N and BFI_O), the Chinese dataset showed significant correlations. The results from the French data, however, showed less divergence from the original results: only the correlation between FW and BFI-C was significant. whereas the original study did not report this effect (see figure 2). These results suggested that the FW dimension may measure a different construct than those in Western cultural context.

For the FD (Fatalistic Determinism) dimension, the Chinese dataset also displayed a different pattern from the original study in two out of three MLOC dimensions and four out of five BFI dimensions. Although most correlations were in the same direction in the Chinese dataset and the original study, the relationship between FD and openness (BFI_O) was reversed: the original study reported a positive correlation, we found a negative correlation instead. The French dataset also showed a similar pattern for the relationships, with one exception. The relationship between FD and agreeableness (BFI_A) as compared to the original study: while the original study reported a positive correlation, the French data showed a non-significant correlation.

For the SD (Scientific Determinism) and UP (Unpredictability) dimensions, we found less divergence between our data and the original study. In the Chinese dataset, SD showed different correlations in three out of five dimensions of BFI, but we did not observe significant differences in the correlations between SD and the three dimensions of MLOC. The UP dimension also showed different correlations in two out of five dimensions of BFI, but we did not observe differences in the correlations for the three dimensions of MLOC. In French data, we did not find differences between SD and any BFI dimensions, but we found the correlation between UP and two out of five dimensions of BFI differed from the original.

### 3.4. Measurement invariance

Table 3 summarizes the outcomes of measurement invariance analyses for the Chinese, French and Japanese datasets when compared to English dataset. We observed only partial measurement invariance (*p* = 0.07) for the Chinese versions compared to the English version, suggesting that the factor loadings may not differ significantly across these cultural contexts. For the Chinese and English comparison (ENG-CHN), the configural model fit the data well, indicating that the basic factor structure was achieved. However, when moving to more constrained models, the weak model (*χ²* = 9197.0, *df* = 659, CFI = 0.784, RMSEA = 0.076) exhibited a decrease in CFI and an increase in RMSEA compared to the configural model, indicating that factor loadings may not be equivalent between these two groups. Similarly, for the French and English comparison (ENG-FRN) and the comparison between the Japanese datasets and the English dataset, the configural model was achieved, but the more constrained models, including the partial model, showed a decline in fit (*p*<0.05). However, as reported in Stage 1 electronic supplementary material, based on the IRT method, the comparison between the Japanese dataset 2 and the English dataset showed partial MI with *p* = 0.011. In line with Stage 1 plan, we used the IRT method to identify items without differential item functioning (DIF) and found that only item SD2 (‘People’s biological makeup determines their talents and personality’) exhibited measurement invariance between the Chinese and English datasets.

**Table 3.** Results of the measurement invariance analyses comparing different languages’ datasets.

ENG-CHN	*χ* ^2^	*df*	CFI	RMSEA	ΔCFI	ΔRMSEA
configural	8299.7	636	0.806	0.073		
partial	10 500 ·	679 (677)	0.751 (0.751)	0.080 (0.080)	0.000	0.000
weak	9197.0***	659	0.784	0.076	0.022	0.003
ENG-FRN	*χ* ^2^	*df*	CFI	RMSEA	ΔCFI	ΔRMSEA
configural	7288.5	636	0.813	0.071		
partial	8854.8*** (8842)	679 (677)	0.770 (0.770)	0.076 (0.076)	0.000	0.000
weak	7418.8***	659	0.810	0.070	0.003	0.001
ENG-JPN_1	*χ* ^2^	*df*	CFI	RMSEA	ΔCFI	ΔRMSEA
configural	11 286	636	0.803	0.069		
partial	13555*** (13 529)	679 (677)	0.762 (0.762)	0.074 (0.074)	0.000	0.000
weak	11986***	659	0.790	0.070	0.013	0.001
ENG-JPN_2	*χ* ^2^	*df*	CFI	RMSEA	ΔCFI	ΔRMSEA
configural	7491.9	636	0.810	0.073		
partial	9271.9* (9262.8)	679 (677)	0.762 (0.762)	0.079 (0.079)	0.000	0.000
weak	7971.0***	659	0.797	0.074	0.013	0.001

*Note* ‘***” *p*<0.005; ‘**” *p*<0.01; ‘*” *p*<0.05; ‘.” *p*<0.1; “ ” *p*<1

We also conducted MI analyses within subgroups of the same languages, specifically within the Chinese and Japanese datasets. We found that three Chinese sub-groups exhibited partial MI, and the two Japanese datasets exhibited partial MI as well. For additional details, see electronic supplementary material, 2.2.

## 4. Discussion

This registered report aimed to examine the measurement invariance of the FAD-Plus scale across four languages. We compared a newly collected Chinese dataset and available Japanese and French data with an open English dataset of FAD-Plus. We found that the four-factor model of FAD-Plus held for the four language datasets. Our multi-group CFA revealed partial measurement invariance between the Chinese and English datasets, as well as between one of the Japanese datasets and the English dataset. The reliability of FAD-Plus was comparable to the original study in all four languages. However, the criterion validity of the FAD-Plus in the Chinese and French datasets showed mixed results when compared to the original study.

Our primary analyses focused on the measurement invariance between the following pairs: Chinese and English, Japanese dataset 1 and English, Japanese dataset 2 and English and French and English. The results revealed that while the four-factor model of FAD-Plus held across all datasets, only weak partial invariance was found between the Chinese-English dataset pair and the Japanese dataset 2 and English dataset pair. For the other two dataset pairs, we did not find partial metric invariance. Our further analyses with IRT revealed that only one item from the Scientific Determinism subscale (SD2, ‘People’s biological makeup determines their talents and personality’) exhibited measurement invariance between the Chinese-English datasets, but none exhibited measurement invariance for the Japanese dataset 2 and English dataset pair. Given that partial metric invariance was only achieved by allowing the factor loadings of 21 out of 27 items to vary across groups, our results suggest near non-invariance of FAD-Plus across different languages and caution when interpreting the score of the FAD-Plus obtained across different languages.

The lack of measurement invariance might have three major sources: item bias, method bias and construct bias [60]. Item bias refers to anomalies at the item level, such as poor translations [61] or the inclusion of terms that have a culture-specific interpretation. Method bias results from differences in the methods used, such as differences in sampling procedures across populations [62,63], differences in non-response patterns [64,65], variations in familiarity with stimuli across groups, and differences in questionnaire administration. Our data suggested that the non-invariance was not due to poor item quality or method bias, as the results did not improve even when we applied multi-group CFA to data from the same language (e.g. datasets from Chinese or Japanese, see electronic supplementary material, results 2.2).

The third source, construct bias, is probably the reason for our near non-invariance results. Construct bias is the most fundamental form of bias and means that the construct itself is interpreted differently across groups [66], which aligns with our reasoning prior to data collection and cross-cultural differences in the interpretation of ‘free will’ [1]. Indeed, our criterion validity results suggest that the constructs measured by the Chinese FAD-Plus may differ from those in the original study. In the original study as well as in other studies conducted in Western culture, free will belief was expected to be moderately correlated to the internal control dimension of MLOC and not necessarily negatively correlated with the powerful other or chance dimension of MLOC, because free will belief and internal control are two distinct constructs [4,16]. However, we found that FW had a strong, rather than moderate, positive correlation with internal control (*r* = 0.577, 95% CI [0.467, 0.675]) and has negative correlations with powerful others (*r* = - 0.288, 95% CI [−0.414,−0.149]) and chance (*r* = −0.324, 95% CI [−0.446,−0.194]). These negative correlations between FW and powerful others, and FW and chance, are similar to those between internal and powerful others (*r* = −0.232, 95% CI [−0.370,−0.084]) and between internal and chance (*r* = −0.392, 95% CI [−0.513,−0.257]), suggesting that FW might be perceived similarly to internal control in Chinese samples, rather than as a distinct construct. Note that our criterion validity results may be caused by the lack of measurement invariance for the BFI/MLOC across different cultural contexts. There was evidence that the BFI−2 (the version we used) was ‘largely invariant’ across Chinese and US samples [43], but the measurement invariance of MLOC is still an open question. These findings suggested that a thorough examination of the measurement invariance of belief in free will measurement requires not only the scale itself but also relevant scales to be validated across diverse cultures.

Given the relatively poor results of the MI of FAD-Plus, we carried out an exploratory analysis using EFA and NMF, as planned (see figure 1). Results from the EFA indicated that the four-factor model fit well, with the exception of item UP20 (‘Luck plays a big role in people’s lives’). These results suggested that the four-factor model of FAD-Plus may remain the best candidate model across all these languages. However, NMF revealed that a four-factor model for the English and French data, the best model for the Chinese and Japanese data is a three-factor model (see §3 of the electronic supplementary material). The slight differences in results for the Chinese and Japanese datasets between EFA and NMF may be attributed to the internal algorithms of each method: EFA assumes normally distributed data and aims to explain the covariance among observed variables; in contrast, NMF is a more data-driven approach that decomposes the data matrix into non-negative factors, identifying the most distinct and clear-cut factors or patterns within the data. The nuanced differences highlighted by NMF suggest that a four-factor model may be acceptable for the Chinese and Japanese datasets, whereas a three-factor model is preferable from a purely data-driven perspective. This lack of convergence between EFA and NMF underscores the importance of integrating various data analysis approaches in cross-cultural studies (e.g. [67]).

Taken together, our results revealed that only partial metric invariance can be achieved for the FAD-Plus across different languages and across different datasets within the same language. These results call attention to the clarity of the four constructs of FAD-Plus and their validity [18,20] and cross-cultural generalizability [1]. The unsatisfactory results of the MI analysis highlight a new area for future research, for example, by combining with new practices such as big-team science [68,69] and adversarial collaboration [70].

More specifically, we suggest that future work could combine top-down (theory-driven) and bottom-up (data-driven) approaches to deepen our understanding of belief in free will. Firstly, an adversarial collaboration (e.g. [71]) is needed for a better conceptualization of free will belief and for determining which dimensions should be measured alongside the belief in free will. Although the configural MI of FAD-Plus seems to be achieved, we should not ignore the fact that there are other free will belief scales that include dimensions such as dualism (FWI [13]). Moreover, recent studies revealed that dimensions such as attitudes towards free will [5] and dualism [14] are closely related to free will beliefs. Thus, a collaborative theory-driven approach is needed to inform a better conceptualization of belief in free will and its nomological networks and construct validity [72]. Adversarial collaboration provides a good opportunity for researchers, with their own conceptual frameworks, to work together and work towards developing a unified framework for measuring belief in free will. Meanwhile, given that previous studies of free will beliefs have largely been Western, it is necessary to include researchers from more diverse cultures to obtain a more generalizable concept of free will from the very beginning.

Secondly, the theory-driven approach should be complemented by a bottom-up approach, in which participants’ interpretations and experiences of the concepts should be elicited and analyzed. For example, to study the positive emotions elicited when experiencing or observing a sudden intensification of communal sharing relationships, Seibt *et al*. [73] tested their theoretical model by presenting videos to participants and asking them to report their feelings on several dimensions. Given the abstractness of free will, it may not be easy to evoke participants’ experiences using videos; however, it is possible to explore beliefs about free will by studying lay people’s intuitions [4] or their prototypes of free will [74]. Implementing bottom-up approaches requires large-scale data collection, which can be achieved through large-scale collaborative science initiatives, such as the Psychological Science Accelerator [75].

In conclusion, our findings shed light on the nuances of measurement invariance in the context of the FAD-Plus scale across different languages. These findings call for studies on the measurement of belief in free will with the state-of-the-art practices such as adversarial collaboration, cross-cultural studies and/or big team science.

## Data Availability

Materials: All materials used for data collection in China are available at [76]. Code and Raw data: All de-identified raw data (in CSV format), and related R scripts are also available at: [77] and [76]. The dataset used in our study were from multiple sources. More specifically, we included several open or shared datasets from the following studies (classification by language): The Chinese dataset were newly collected and are available at [76]. The English dataset was from three recent papers: [36–38]. The French dataset wass from: [17]. The Japanese dataset was from two papers: [19,23]. Supplementary material is available online [78].
